# Detection of Elusive Rogue Wave with Cross-Track Interferometric Synthetic Aperture Radar Imaging Approach

**DOI:** 10.3390/s25092781

**Published:** 2025-04-28

**Authors:** Tung-Cheng Wang, Jean-Fu Kiang

**Affiliations:** Graduate Institute of Communication Engineering, National Taiwan University, Taipei 10617, Taiwan

**Keywords:** rogue wave, probability-based model, cross-track interferometric SAR (XTI-SAR), satellite altimetry

## Abstract

Rogue waves are reported to wreck ships and claim lives. The prompt detection of their presence is difficult due to their small footprint and unpredictable emergence. The retrieval of sea surface height via remote sensing techniques provides a viable solution for detecting rogue waves. However, conventional synthetic aperture radar (SAR) techniques are ineffective at retrieving the surface height profile of rogue waves in real time due to nonlinearity between surface height and normalized radar cross-section (NRCS), which is not obvious in the absence of rogue waves. In this work, a cross-track interferometric SAR (XTI-SAR) imaging approach is proposed to detect elusive rogue waves over a wide area, with sea-surface profiles embedding rogue waves simulated using a probability-based model. The performance of the proposed imaging approach is evaluated in terms of errors in the position and height of rogue-wave peaks, the footprint area of rogue waves, and a root-mean-square error (RMSE) of the sea-surface height profile. Different rogue-wave events under different wind speeds are simulated, and the reconstructed height profiles are analyzed to determine the proper ranges of look angle, baseline, and mean-filter size, among other operation variables, in detecting rogue waves. The proposed approach is validated by simulations in detecting a rogue wave at a spatial resolution of 3 m × 3 m and height accuracy of decimeters.

## 1. Introduction

Rogue waves are notorious for their unpredictable emergence in many different water bodies [1]. They are believed to have accounted for hundreds of incidents over the past decades, claiming lives and causing ship damage [1,2,3]. The scarcity of in situ measurement data is attributed to their small footprint, short bursts, and unpredictable moment and site of emergence [4,5]. On 1 January 1995, a rogue wave with height of 25.6 m was recorded for the first time near Draupner Jacket platform, North Sea [6], which was later referred to as the Draupner wave or the New Year’s wave [7].

Remote sensing techniques with real-time response over a vast area are needed to accurately detect elusive rogue waves. Synthetic aperture radar (SAR) is a viable technique for monitoring vast ocean areas, without being sabotaged by cloud cover or solar insolation. In [8,9], a set of SAR images embedding rogue-wave events were simulated, in which the perturbation of normalized radar cross section (NRCS) was exploited to detect rogue-wave events. In [10], numerical simulations implied that the NRCS of rogue waves is significantly smaller than the background waves, and an NRCS threshold was recommended to locate rogue waves. However, NRCS only serves as an indirect indicator of rogue waves. The European Commission funded the MaxWave project to detect rogue waves by estimating the surface height from the variation of normalized radar cross-sections [5]. However, the height derived from NRCS is nonlinearly related to the surface height [11], rendering SAR techniques inaccurate in retrieving the surface height profile of rogue waves.

The sea-surface height profile has been measured with conventional altimeters by emitting nadir pulses [12], rendering km resolution, which is insufficient for rogue-wave detection. SAR altimetry combining conventional altimeters and SAR techniques can improve the horizontal resolution to several hundred meters and has been applied to detect internal waves [13].

Meanwhile, cross-track interferometric SAR (XTI-SAR) has been widely used to retrieve the terrain surface height from the phase difference of radar echoes at two separate radar receivers [14], which is less sensitive to the variation of NRCS. XTI-SAR systems have been used to build a digital elevation model [15] and to detect surface deformations [16]. In [17], an airborne Ka-band XTI-SAR system was demonstrated to acquire glacier and ice surface topography, with a height accuracy of a few decimeters and spatial resolution of 100 m. In [18], a Ku-band long-baseline (about 1000 m) imaging radar altimetry (IRA), inspired by the TanDEM-X satellite formation, was proposed to improve the height accuracy to about 1 cm. A helix formation of two satellites was configured, by properly adjusting their orbital inclination and eccentricity [19], to conduct wide-swath ocean interferometric altimetry [20].

SAR altimetry, as a variation of the XTI-SAR system, also exploits the phase difference of radar echoes to acquire sea-surface height information. A near-nadir look angle was chosen by referring to the nadir operation of conventional altimeters [21]. For example, the Surface Water and Ocean Topography (SWOT) mission was launched in December 2022 to provide a high-resolution water elevation map [22], which has found various oceanographic applications, including seafloor topography retrieval [23], sea-surface anomaly due to cyclone [24], and inland water surface elevation retrieval [25]. Another example is the Tiangong-2 space laboratory, which can conduct internal-wave signature retrieval [26] and marine gravity recovery [27]. However, the layover effect at near-nadir incidence impeded the detection of rogue waves, which manifest drastic height changes over a short horizontal extent, making it unsuitable for rogue wave detection.

Thus, an XTI-SAR system was proposed for detecting rogue waves in this work, which provides high spatial resolution and height accuracy to capture waves with high steepness and abrupt height changes. As far as we know, no XTI-SAR system has been customized for detecting rogue waves.

Limited by the scarcity of measurement data on rogue waves, the proposed XTI-SAR imaging technique will be verified by reconstructing simulated rogue waves. Various mechanisms underpinning rogue waves have been studied, including linear addition [28], wave-current interaction, nonlinear focusing [29], modulational instability, and soliton collision [4]. The evolution of rogue waves can be described by using the nonlinear Schrödinger equation (NLSE) [30], Sasa-Satsuma equation (SSE) [31], Hirota equation [32], modified Korteweg-de Vries (mKdV) equation [33], and so on. The solutions of these differential equations bear different features that may deviate from those of observed rogue waves [34]. Furthermore, a nonlinear model based on differential equations is unable to represent the random nature of ocean waves, making it less effective in simulating a large number of rogue wave events.

In [8,35], a linear-wave focusing technique was proposed, by imposing random phases in multiple linear wave components, to generate high-amplitude spikes out of constructive interference, mimicking temporal Draupner waves [10,36]. Its efficacy was verified by comparing the spectrum of 1D sea-surface profiles with field measurement data. In [37], a rogue wave was synthesized by superposing a random wave train and a focusing wave train, with both wave trains formed by the linear superposition of plane waves, exhibiting second-order interactions among the components [38]. The idea was extended to synthesize two-dimensional rogue waves [34] and is adopted in this work to simulate the surface profiles of rogue waves.

In this work, an XTI-SAR imaging approach is proposed to directly retrieve the sea-surface height of two-dimensional rogue waves, which are simulated by superposing a random wave train and a focusing wave train at a high emergence rate of rogue waves (83%), facilitating the generation of a plethora of rogue waves for statistical analysis and image reconstruction. Many realizations of rogue waves are simulated under different wind speeds to study the statistical characteristics of such events and to test the performance of the proposed XTI-SAR imaging approach. The layover effect associated with the steepness of rogue waves is analyzed, leading to the conclusion that near-nadir incidence is ineffective in detecting rogue waves, and a minimum look angle is required to reconstruct the height profile of a rogue wave. Geometric correction is applied to fix the location of rogue-wave peaks, which can be severely misplaced without correction. The peak shift caused by the steepness of rogue waves is reduced from 10 m to about 1 m. Proper radar parameters, including look angle, baseline, and mean-filter size are fine-tuned and justified via the quality of reconstructed images, in terms of errors in rogue-wave peak position and height, rogue wave footprint size, and root-mean-square error (RMSE) of the image, with three sets of parameters customized for detecting three types of rogue wave. The proposed imaging approach is validated by simulations in detecting a rogue wave at a spatial resolution of 3 m × 3 m and a height accuracy of decimeters.

The rest of this work is organized as follows. The synthesis of rogue waves is presented in Section 2, the XTI-SAR imaging approach is presented in Section 3, the simulations are discussed in Section 4, and some conclusions are drawn in Section 5.

## 2. Synthesis of Rogue Waves

**Figure 1:**
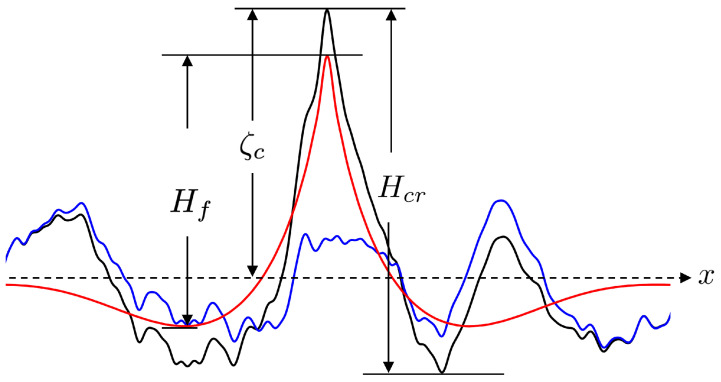
Snapshot of sea-surface profile (**———**) along *x*-axis, −−−: mean sea level, **———**: random wave train, **———**: focusing wave train.

A rogue wave owns conspicuous features in its temporal waveform and spatial image [4]. Figure 1 shows a snapshot of a sea-surface profile along the *x*-axis. A rogue wave is designated as such if its wave height $H_{cr}$ satisfies [4]

(1) $$D_{1}:\mathrm{AI}=H_{cr}/H_{1/3}>2$$

where AI is the abbreviation of abnormality index and $H_{1/3}$ is the significant wave height of the surface-height profile $\zeta [n_{r},n_{a}]$, with $1\leq n_{r}\leq N_{r}$ in range and $1\leq n_{a}\leq N_{a}$ in azimuth, which can be approximated as

(2) $$\begin{array}{ccc} & & H_{1/3}≃4\sqrt{\frac{1}{N_{r}N_{a}}\sum_{n_{r}=1}^{N_{r}}\sum_{n_{a}=1}^{N_{a}}\zeta^{2}\left[n_{r},n_{a}\right]} \end{array}$$

A directional JONSWAP spectrum is adopted to simulate sea-surface profiles, which is given by [39]

(3) $$\begin{array}{ccc} & & \Psi (\omega ,\theta )=S(\omega )\Phi (\theta ) \end{array}$$

where θ is the azimuth direction,

(4) $$\begin{array}{ccc} & & \Phi \left(\theta \right)=\left\{\begin{array}{cc} \frac{2}{\pi }\cos^{2}\left(\theta -\theta_{w}\right),\;\; & |\theta -\theta_{w}|\leq \frac{\pi }{2},\\ 0, & \mathrm{otherwise} \end{array}\right. \end{array}$$

is an angular spreading function about the wind direction $\theta_{w}$ [39], with

$$\begin{array}{ccc} & & \int_{-\pi }^{\pi }\Phi \left(\theta \right)d\theta =1 \end{array}$$

The omni-directional JONSWAP spectrum in (3) is given by [40]

(5) $$\begin{array}{ccc} & & S\left(\omega \right)=\frac{\alpha g^{2}}{\omega^{5}}\exp\left\{-\frac{5}{4}{\left(\frac{\omega_{p}}{\omega }\right)}^{4}\right\}\gamma^{\Gamma (\omega )} \end{array}$$

where γ is a peak enhancement factor, $\omega_{p}$ is a peak angular frequency, α is an energy scale factor, *g* is the gravitational acceleration, and

$$\begin{array}{ccc} & & \Gamma \left(\omega \right)=\exp\left\{-\frac{1}{2\sigma^{2}}{\left(\frac{\omega }{\omega_{p}}-1\right)}^{2}\right\} \end{array}$$

with

$$\begin{array}{ccc} & & \sigma =\left\{\begin{array}{cc} 0.07,\;\; & \omega <\omega_{p},\\ 0.09, & \omega >\omega_{p} \end{array}\right. \end{array}$$

The significant wave height $H_{1/3}$ and the energy scale factor α can be represented by polynomial functions of γ [40].

A time-varying sea-surface profile $\zeta (\bar{r},t)$, at $\bar{r}=\hat{x}x+\hat{y}y$, can be approximated as a linear superposition of multiple plane waves as

(6) $$\begin{array}{ccc} & & \zeta \left(\bar{r},t\right)=\sum_{n=1}^{N_{\theta }}\sum_{m=1}^{N_{\omega }}\zeta_{mn}\cos\left(\omega_{m}t-{\bar{k}}_{mn}\cdot \bar{r}+\phi_{mn}\right) \end{array}$$

where $\phi_{mn}$ is a uniform random variable over [0,2π],

$$\begin{array}{ccc} & & {\bar{k}}_{mn}=\hat{x}k_{m}\cos\theta_{n}+\hat{y}k_{m}\sin\theta_{n}\\ & & \omega_{m}=\left(m-1/2\right)\Delta \omega \\ & & \theta_{n}=-\pi /2+\left(n-1/2\right)\Delta \theta \end{array}$$

with $\Delta \omega =5\omega_{p}/N_{\omega }$ and $\Delta \theta =\pi /N_{\theta }$. Each plane wave satisfies the dispersion relation $\omega_{m}^{2}=gk_{m}$ [39], and the amplitude is $\zeta_{mn}=\sqrt{2\Psi (\omega_{m},\theta_{n})\Delta \omega \Delta \theta }$.

The wave height *H* follows a Rayleigh distribution, with a probability density function (PDF) of [41]

$$\begin{array}{ccc} & & P\left(H\right)=\frac{4H}{H_{1/3}^{2}}\exp\left\{-\frac{2H^{2}}{H_{1/3}^{2}}\right\} \end{array}$$

The probability that the wave height satisfies the criterion $D_{1}$ in (1) is

$$\begin{array}{ccc} & & P_{D_{1}}=\int_{2H_{1/3}}^{\infty }P\left(H\right)dH≃3.35\times 10^{-4} \end{array}$$

which is extremely low.

The emergence rate of rogue waves can be boosted in the simulations by implementing a linear focusing method [42], in which the sea-surface profile is approximated by superposing a random wave train $\zeta_{r}$ and a focusing wave train $\zeta_{f}$ as [34]

(7) $$\begin{array}{ccc} & & \zeta \left(\bar{r},t\right)=\zeta_{r}\left(\bar{r},t\right)+\zeta_{f}\left(\bar{r},t\right) \end{array}$$

The random wave train is given by

$$\begin{array}{ccc} & & \zeta_{r}\left(\bar{r},t\right)=\sum_{n=1}^{N_{\theta }}\sum_{m=1}^{N_{\omega }}\zeta_{rmn}\cos\left(\omega_{m}t-{\bar{k}}_{mn}\cdot \bar{r}+\phi_{mn}\right) \end{array}$$

with $\zeta_{rmn}=\sqrt{2p_{r}\Psi \left(\omega_{m},\theta_{n}\right)\Delta \omega \Delta \theta }$. The focusing wave train, centered at location ${\bar{r}}_{c}=\left(x_{c},y_{c}\right)$ and time $t_{c}$, is given by

$$\begin{array}{ccc} & & \zeta_{f}\left(\bar{r},t\right)=\sum_{n=1}^{N_{\theta }}\sum_{m=1}^{N_{\omega }}\zeta_{fmn}\cos\left[\omega_{m}\left(t-t_{c}\right)-{\bar{k}}_{mn}\cdot \left(\bar{r}-{\bar{r}}_{c}\right)\right] \end{array}$$

with $\zeta_{fmn}=\sqrt{2p_{f}\Psi \left(\omega_{m},\theta_{n}\right)\Delta \omega \Delta \theta }$. The fractions of total energy carried by random wave train and focusing wave train are $p_{r}$ and $p_{f}$, respectively, with $p_{r}+p_{f}=1$.

To estimate the focusing wave height $H_{f}$, we assume the propagating direction of the rogue wave is the same as the wind blowing direction, namely, ${\hat{r}}_{w}=\hat{x}\cos\phi_{w}+\hat{y}\sin\phi_{w}$. The wavelength of a rogue wave is approximated as $\lambda_{p}=2\pi g/\omega_{p}^{2}$, where $\omega_{p}$ is the peak angular frequency. The lowest height next to the rogue-wave peak appears at $\bar{r}={\bar{r}}_{c}-{\hat{r}}_{w}\lambda_{p}/2$ and $t=t_{c}$, with a height of $\zeta_{f}\left({\bar{r}}_{c}-{\hat{r}}_{w}\lambda_{p}/2,t_{c}\right)$. Thus, the focusing wave height is approximated as

$$\begin{array}{ccc} & & H_{f}=\zeta_{f}\left({\bar{r}}_{c},t_{c}\right)-\zeta_{f}\left({\bar{r}}_{c}-{\hat{r}}_{w}\lambda_{p}/2,t_{c}\right)\\ & & =\sum_{n=1}^{N_{\theta }}\sum_{m=1}^{N_{\omega }}\sqrt{2p_{f}\Psi \left(\omega_{m},\theta_{n}\right)\Delta \omega \Delta \theta }\left[1-\cos\left({\bar{k}}_{mn}\cdot {\hat{r}}_{w}\lambda_{p}/2\right)\right] \end{array}$$

Similarly, the random wave height is given by

$$\begin{array}{ccc} & & H_{r}=\zeta_{r}\left({\bar{r}}_{c},t_{c}\right)-\zeta_{r}\left({\bar{r}}_{c}-{\hat{r}}_{w}\lambda_{p}/2,t_{c}\right) \end{array}$$

with

(8) $$\begin{array}{ccc} & & \zeta_{r}\left({\bar{r}}_{c},t_{c}\right)=\sum_{n=1}^{N_{\theta }}\sqrt{\Phi (\theta_{n})\Delta \theta }\zeta_{rn}\left({\bar{r}}_{c},t_{c}\right) \end{array}$$

and

(9) $$\begin{array}{ccc} & & \zeta_{rn}\left(\bar{r},t\right)=\sum_{m=1}^{N_{\omega }}\sqrt{2p_{r}S\left(\omega_{m}\right)\Delta \omega }\cos\left(\omega_{m}t-{\bar{k}}_{mn}\cdot \bar{r}+\phi_{mn}\right) \end{array}$$

which resembles a Gaussian random variable [39]. Its variance is related to the zeroth moment of $\zeta_{rn}\left({\bar{r}}_{c},t_{c}\right)$ [34], which is $p_{r}m_{0}$, with $m_{0}=\int_{0}^{\infty }S\left(\omega \right)d\omega$. In other words,

$$\begin{array}{ccc} & & \zeta_{rn}\left({\bar{r}}_{c},t_{c}\right)∼N\left(0,p_{r}m_{0}\right) \end{array}$$

and (8) implies

(10) $$\begin{array}{ccc} & & \zeta_{r}\left({\bar{r}}_{c},t_{c}\right)∼N\left(0,p_{r}m_{0}\sum_{n=1}^{N_{\theta }}\Phi \left(\theta_{n}\right)\Delta \theta \right)=N\left(0,p_{r}m_{0}\right) \end{array}$$

By applying the same argument to $\zeta_{r}\left({\bar{r}}_{c}-{\hat{r}}_{w}\lambda_{p}/2,t_{c}\right)$, the random wave height is reduced to

(11) $$\begin{array}{ccc} & & H_{r}∼N\left(0,2p_{r}m_{0}\right) \end{array}$$

Following the criterion $D_{1}$ in (1), a rogue wave emerges with the probability

(12) $$\begin{array}{ccc} & & P_{rw}=P\left(H>2H_{1/3}\right)=P\left(H_{r}>2H_{1/3}-H_{f}\right)\\ & & =\int_{2H_{1/3}-H_{f}}^{\infty }\frac{1}{2\sqrt{\pi p_{r}m_{0}}}\exp\left\{-\frac{x^{2}}{4p_{r}m_{0}}\right\}dx\\ & & =\frac{1}{2}-\frac{1}{2}\mathrm{erf}\left(\frac{2H_{1/3}-H_{f}}{2\sqrt{p_{r}m_{0}}}\right) \end{array}$$

where $H_{1/3}≃4\sqrt{p_{r}m_{0}}$ [34] and erf(x) is the error function.

## 3. XTI-SAR Imaging Approach

**Figure 2:**
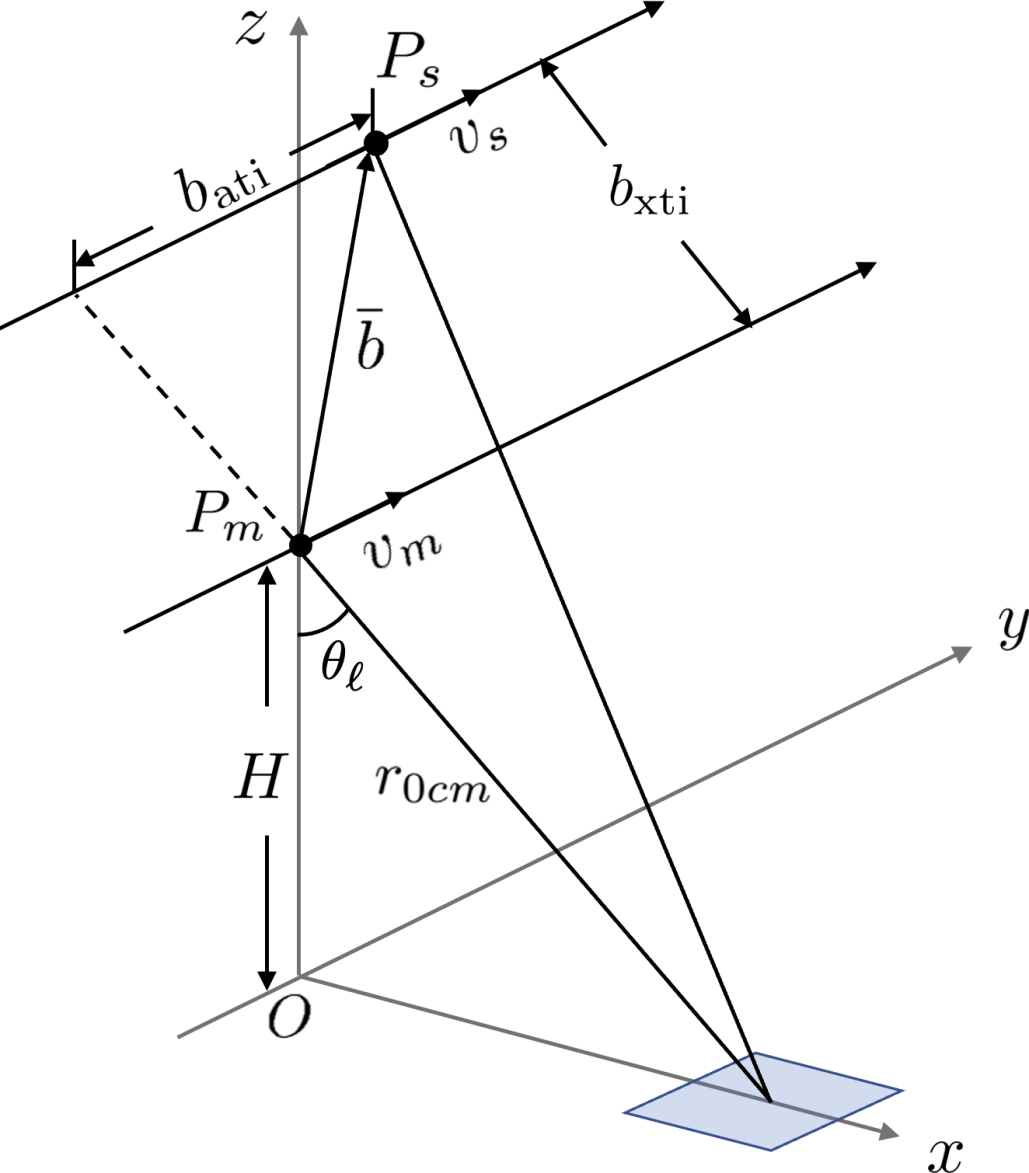
Schematic of single-pass XTI-SAR imaging with master satellite $P_{m}$ and slave satellite $P_{s}$.

Figure 2 shows the schematic of single-pass XTI-SAR imaging, implemented with a master satellite ($P_{m}$) and a slave satellite ($P_{s}$) moving in parallel [14]. The origin is set at the nadir point of the master satellite at η=0, with the *z* direction pointing upwards. The master satellite flies in the *y* direction, at a constant altitude *H*, and the squint angle is set to zero without loss of generality.

The master satellite and the slave satellite are separated by a baseline vector $\bar{b}={\left[b_{x},b_{y},b_{z}\right]}^{t}$, with a cross-track baseline of $b_{\mathrm{xti}}=\sqrt{b_{x}^{2}+b_{z}^{2}}$ and an along-track baseline of $b_{\mathrm{ati}}=b_{y}$, which is set to zero. The look angle measured from the master satellite is $\theta_{l}$. The sea-surface profile is given by z=ζ(x,y), which is modeled as a connected set of tilted facets in computing the backscattered radar signals.

The closest range from the master satellite to the $n_{r}$-th range cell is $r_{0m}\left[n_{r}\right]=c\tau \left[n_{r}\right]/2$, where $\tau \left[n_{r}\right]=2r_{0cm}/c+\left(n_{r}-1-N_{r}/2\right)\Delta \tau$ and $r_{0cm}=r_{0m}\left[N_{r}/2+1\right]=H/\cos\theta_{l}$ is the closest range between the master satellite and the swath center, at $\tau_{0}=2r_{0cm}/c$.

**Figure 3:**
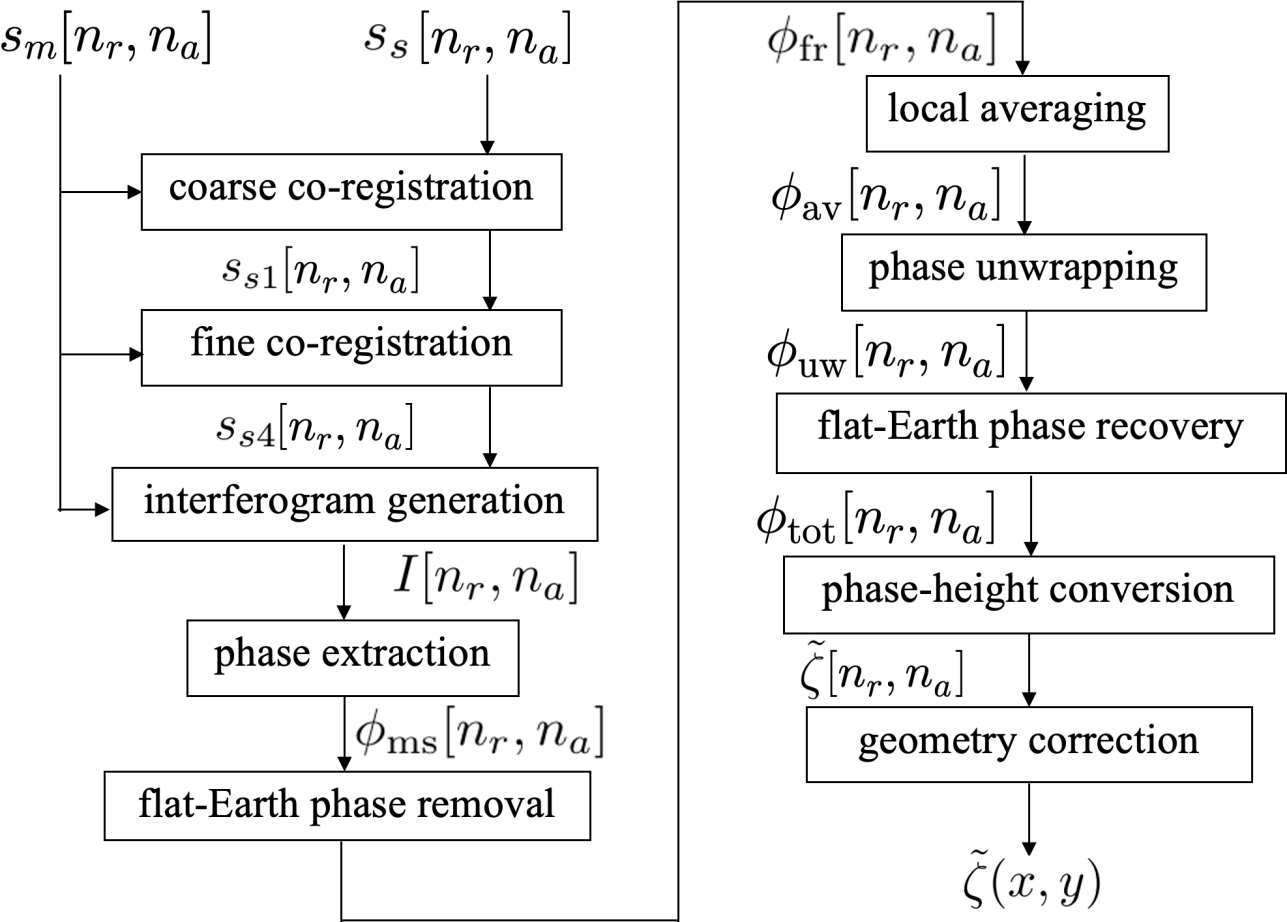
Flow-chart of XTI-SAR imaging algorithm.

Figure 3 shows the flow-chart of the XTI-SAR imaging algorithm for acquiring the sea-surface profile [43].

### 3.1. Co-Registration

The master image and the slave image are registered as $s_{m}\left[n_{r},n_{a}\right]$ and $s_{s}\left[n_{r},n_{a}\right]$, respectively, with $1\leq n_{r}\leq N_{r}$ and $1\leq n_{a}\leq N_{a}$. The slave image is first shifted and resampled via a coarse co-registration process. Each pixel of the slave image is shifted to the right by $\Delta n_{r}$ pixels and to the top by $\Delta n_{a}$ pixels, where $\Delta n_{r}$ and $\Delta n_{a}$ are determined by maximizing a correlation function [44]

$$\begin{array}{ccc} & & \;\left(\Delta n_{r},\Delta n_{a}\right)=\arg\;\max_{\left(p,q\right)}\gamma_{pq}\left\{s_{m}\left[n_{r},n_{a}\right],s_{s}\left[n_{r},n_{a}\right]\right\} \end{array}$$

with

(13) $$\begin{array}{ccc} & & \;\gamma_{pq}\left\{s_{m}\left[n_{r},n_{a}\right],s_{s}\left[n_{r},n_{a}\right]\right\}=\frac{\left|E\{s_{m}\left[n_{r},n_{a}\right]s_{s}^{*}\left[n_{r}+p,n_{a}+q\right]\}\right|}{\sqrt{E\{|s_{m}\left[n_{r},n_{a}\right]{|}^{2}\}E\{|s_{s}\left[n_{r}+p,n_{a}+q\right]{|}^{2}\}}} \end{array}$$

where E(x) is the expectation value of *x*. The slave image after coarse co-registration is registered as

(14) $$\begin{array}{ccc} & & s_{s1}\left[n_{r},n_{a}\right]=s_{s}\left[n_{r}+\Delta n_{r},n_{a}+\Delta n_{a}\right] \end{array}$$

Next, a fine co-registration process is conducted by first oversampling the slave image $s_{s1}\left[n_{r},n_{a}\right]$ by a factor $N_{s}$ in the range direction [45] to acquire an upsampled image $s_{s3}\left[n_{r},n_{a}\right]$. Then, $s_{s3}\left[n_{r},n_{a}\right]$ is segmented into $N_{rt}\times N_{at}$ sub-images, with each containing $N_{rp}\times N_{ap}$ pixels, namely,

(15) $$\begin{array}{ccc} & & \;s_{s3pq}\left[n_{r},n_{a}\right]=s_{s3}\left[n_{r}+\left(p-1\right)N_{rp},n_{a}+\left(q-1\right)N_{ap}\right],\\ & & \;1\leq n_{r}\leq N_{rp},1\leq n_{a}\leq N_{ap} \end{array}$$

Similarly, the master image is segmented into $N_{rt}\times N_{at}$ sub-images, with

$$\begin{array}{ccc} & & \;s_{mpq}\left[n_{r},n_{a}\right]=s_{m}\left[n_{r}+\left(p-1\right)N_{rp},n_{a}+\left(q-1\right)N_{ap}\right],\\ & & \;1\leq n_{r}\leq N_{rp},1\leq n_{a}\leq N_{ap} \end{array}$$

Then, the correlation functions defined in (13) are applied to determine the fine shifts of $s_{s3pq}$ as

$$\begin{array}{ccc} & & \;\left(\Delta n_{rf}^{\left(pq\right)},\Delta n_{af}^{\left(pq\right)}\right)=\arg\;\max_{\left(u,v\right)}\gamma_{uv}\left(s_{mpq}\left[n_{r},n_{a}\right],s_{s3pq}\left[n_{r}^{\prime },n_{a}\right]\right) \end{array}$$

with $n_{r}^{\prime }=1+\left(n_{r}-1\right)N_{s}$, to derive the co-registered slave sub-images as

(16) $$\begin{array}{ccc} & & \;s_{s4pq}\left[n_{r},n_{a}\right]=s_{s3pq}\left[n_{r}^{\prime }+\Delta n_{rf}^{\left(pq\right)},n_{a}+\Delta n_{af}^{\left(pq\right)}\right],\\ & & \;1\leq n_{r}\leq N_{rp},1\leq n_{a}\leq N_{ap} \end{array}$$

Finally, the co-registered slave sub-images are concatenated to form the co-registered slave image $s_{s4}\left[n_{r},n_{a}\right]$.

### 3.2. Removal of Flat-Earth Phase

The interferogram is computed as

(17) $$\begin{array}{ccc} & & I\left[n_{r},n_{a}\right]=s_{m}\left[n_{r},n_{a}\right]s_{s4}^{*}\left[n_{r},n_{a}\right] \end{array}$$

from which the interferometric phase is extracted as

(18) $$\begin{array}{ccc} & & \phi_{\mathrm{ms}}\left[n_{r},n_{a}\right]=\arg\left\{I\left[n_{r},n_{a}\right]\right\} \end{array}$$

To remove the flat-Earth phase that overwhelms the surface-profile information, the closest range between the slave satellite to the $n_{r}$-th range cell is first calculated as

$$\begin{array}{ccc} & & r_{0s}\left[n_{r}\right]={\left\{{\left(r_{0m}\left[n_{r}\right]\right)}^{2}+b^{2}-2br_{0m}\left[n_{r}\right]\cos\left(\frac{\pi }{2}-\theta_{l0}\left[n_{r}\right]+\theta_{b}\right)\right\}}^{1/2} \end{array}$$

where $b=|\bar{b}|$ and $\theta_{b}=\cos^{-1}\left(b_{x}/b\right)$, and $\theta_{l0}\left[n_{r}\right]=\cos^{-1}\left(H/r_{0m}\left[n_{r}\right]\right)$ is the local look angle.

The flat-Earth phase is

(19) $$\begin{array}{ccc} & & \phi_{\mathrm{fe}}\left[n_{r}\right]=-\left(4\pi /\lambda \right)\left(r_{0m}\left[n_{r}\right]-r_{0s}\left[n_{r}\right]\right) \end{array}$$

which is removed from (18) to give

(20) $$\begin{array}{ccc} & & \phi_{\mathrm{fr}}\left[n_{r},n_{a}\right]=\phi_{\mathrm{ms}}\left[n_{r},n_{a}\right]-\phi_{\mathrm{fe}}\left[n_{r}\right] \end{array}$$

### 3.3. Phase Unwrapping and Mean Filter

To mitigate phase noises brought in by the previous steps, a mean filter of size $N_{wr}\times N_{wa}$ is exerted on $\phi_{\mathrm{fr}}\left[n_{r},n_{a}\right]$ to derive [43]

$$\begin{array}{ccc} & & \phi_{\mathrm{av}}\left[n_{r},n_{a}\right]=\arg\left\{\sum_{n=-(N_{wa}-1)/2}^{(N_{wa}-1)/2}\sum_{m=-(N_{wr}-1)/2}^{(N_{wr}-1)/2}e^{j\phi_{\mathrm{fr}}\left[n_{r}+m,n_{a}+n\right]}\right\} \end{array}$$

To explicate the phase unwrapping process, a wrapping operator is first defined as [46]

$$\begin{array}{ccc} & & W\left(\phi \right)=\phi -2\pi \left\lfloor \frac{\phi +\pi }{2\pi }\right\rfloor \end{array}$$

which folds phase ϕ into the interval (−π,π]. Next, the difference between phase $\phi_{\mathrm{av}}\left[n_{r}+1,n_{a}+1\right]$ and its linear approximation ${\tilde{\phi }}_{\mathrm{av}}\left[n_{r}+1,n_{a}+1\right]$ is computed as [46]

$$\begin{array}{ccc} & & t\left[n_{r},n_{a}\right]=\left|W({\tilde{\phi }}_{\mathrm{av}}\left[n_{r}+1,n_{a}+1\right]-\phi_{\mathrm{av}}\left[n_{r}+1,n_{a}+1\right])\right| \end{array}$$

Then, a quality function is derived as [47]

(21) $$\begin{array}{ccc} & & Q\left[n_{r},n_{a}\right]=1-\frac{t[n_{r},n_{a}]}{\pi } \end{array}$$

which falls in [0,1].

A quality-guided phase unwrapping algorithm based on the quality function in (21) is applied [48]. The pixel with the highest quality function in the whole image is selected as the starting point, and its four neighboring pixels are stored in a list. Next, the pixel *p* with the highest quality function is selected from the list, and its unwrapped phase is computed as

$$\begin{array}{ccc} & & \phi_{w}+2\pi \left\lfloor \frac{\phi_{r}-\phi_{w}+\pi }{2\pi }\right\rfloor \end{array}$$

where $\phi_{w}$ is its wrapped phase and $\phi_{r}$ is the unwrapped phase of a neighboring pixel. Pixel *p* is then removed from the list, and its neighboring pixels, which have not been unwrapped, are added to the list. The procedure continues until the list is empty. The unwrapped interferometric phase is registered as $\phi_{\mathrm{uw}}\left[n_{r},n_{a}\right]$.

### 3.4. Surface Height Mapping and Geometric Correction

**Figure 4:**
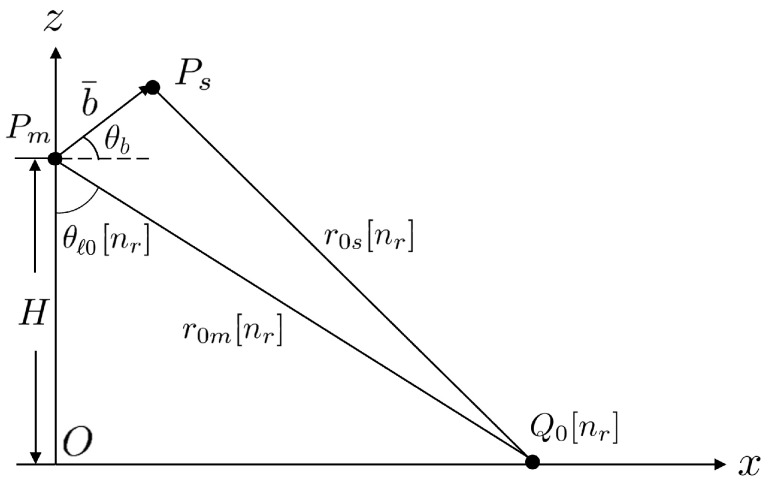
Schematic of height estimation.

Figure 4 shows the schematic to estimate the height at pixel $\left[n_{r},n_{a}\right]$. Before estimating the surface height, the flat-Earth phase in (19) is added back to the unwrapped phase $\phi_{\mathrm{uw}}\left[n_{r},n_{a}\right]$ to derive the total phase

(22) $$\begin{array}{ccc} & & \phi_{\mathrm{tot}}\left[n_{r},n_{a}\right]=\phi_{\mathrm{uw}}\left[n_{r},n_{a}\right]+\phi_{\mathrm{fe}}\left[n_{r}\right] \end{array}$$

from which the range difference $\delta r[n_{r},n_{a}]$ is derived as

(23) $$\begin{array}{ccc} & & \phi_{\mathrm{tot}}\left[n_{r},n_{a}\right]≃\frac{4\pi \delta r[n_{r},n_{a}]}{\lambda } \end{array}$$

By applying the law of cosines, under the assumption that $r_{0m}\gg \delta r$ and $r_{0m}\gg b$, we have

(24) $$\begin{array}{ccc} & & \theta_{l\zeta }\left[n_{r},n_{a}\right]≃\theta_{b}-\frac{\pi }{2}+\cos^{-1}\frac{\delta r[n_{r},n_{a}]}{b} \end{array}$$

The surface height $\zeta [n_{r},n_{a}]$ is then estimated as

(25) $$\begin{array}{ccc} & & \tilde{\zeta }\left[n_{r},n_{a}\right]=H-r_{0m}\left[n_{r}\right]\cos\theta_{l\zeta }\left[n_{r},n_{a}\right] \end{array}$$

**Figure 5:**
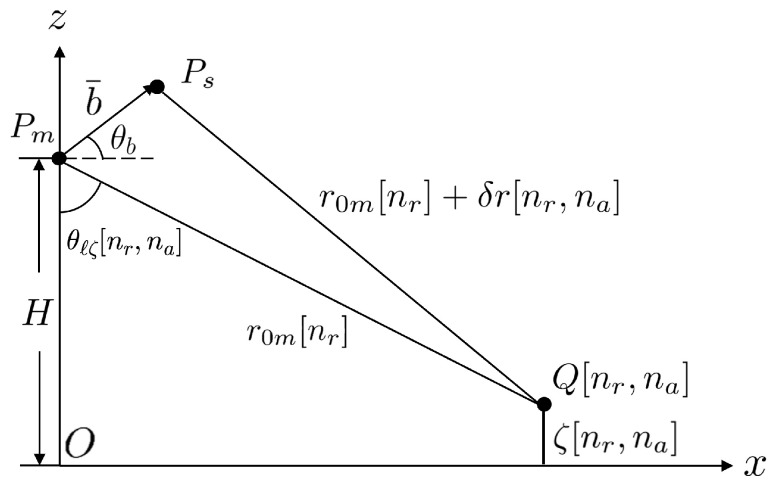
Schematic of geometric correction.

As an uneven surface is mapped to a two-dimensional XTI-SAR image, errors in horizontal coordinates occur and need to be corrected. Figure 5 shows the schematic of geometric correction. A point $Q[n_{r},n_{a}]$ on the surface is mapped to $Q_{0}\left[n_{r},n_{a}\right]$ on the XTI-SAR image, with $\bar{P_{m}Q_{0}}=\bar{P_{m}Q}$, namely,

$$\begin{array}{ccc} & & {\left(H\tan\theta_{l0}+\delta x\right)}^{2}+{\left(H-\tilde{\zeta }\right)}^{2}=r_{0m}^{2} \end{array}$$

where $\delta x[n_{r},n_{a}]$ is the error in the horizontal coordinate, which is estimated as

(26) $$\begin{array}{ccc} & & \delta x≃\tilde{\zeta }\cot\theta_{l0} \end{array}$$

Thus, the horizontal coordinates of image pixel $\left[n_{r},n_{a}\right]$ are corrected as

$$\begin{array}{ccc} & & x=\delta x\left[n_{r},n_{a}\right]+\sqrt{{\left(c\tau [n_{r}]/2\right)}^{2}-H^{2}}\\ & & y=v_{s}\eta \left[n_{a}\right] \end{array}$$

## 4. Simulations and Discussions

The Draupner wave, or the New Year’s wave, has been extensively studied since its emergence [6], including weather condition and ocean environment [49], its possible mechanism [38], numerical simulations [50], and laboratory reconstruction [7]. In [50], the JONSWAP spectrum was used to reconstruct the random sea surface that fostered the New Year’s wave, with relevant parameters estimated from the meteorological condition sand the wind speed $U_{10}$ estimated from $H_{1/3}$. The JONSWAP parameters used to reproduce the New Year’s wave are listed in case 1 of Table 1.

To investigate the effect of wind speed, the JONSWAP parameters associated with $U_{10}=13.7$ m/s at the upper bound of Beaufort scale 6 and $U_{10}=7$ m/s with $H_{1/3}=1$ m are listed in cases 2 and 3, respectively, of Table 1, while the focusing wave ratio is fixed at $p_{f}=0.01$. To investigate the effect of rogue-wave shape, the JONSWAP parameters associated with $p_{f}=0.03$ and 0.1 are listed in cases 4 and 5, respectively, of Table 1, while $U_{10}$ is fixed at 7 m/s.

**Figure 6:**
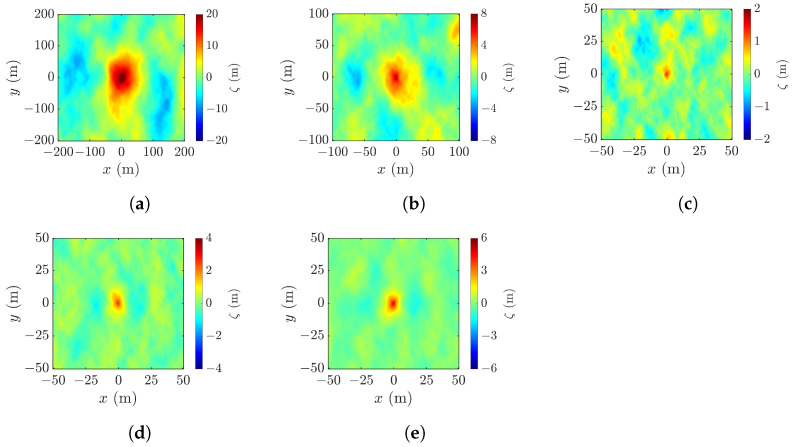
Images of simulated rogue wave with parameters listed in Table 1: (**a**) case 1, (**b**) case 2, (**c**) case 3, (**d**) case 4, (**e**) case 5.

Figure 6 shows the realizations of rogue waves simulated by using the parameters listed in Table 1. Figure 6a reproduces the Draupner event, with the highest point appearing at $\left(x_{c},y_{c},\zeta_{c}\right)=\left(0,-1,23.56\right)$ m, the wave height $H_{\mathrm{cr}}=32.58$ m, and the abnormality index AI =2.4033>2, meeting the criterion $D_{1}$ in (1). Figure 6b shows a realization under strong wind, with the highest point at $\left(x_{c},y_{c},\zeta_{c}\right)=\left(0,0,7.08\right)$, the wave height $H_{\mathrm{cr}}=10.45$ m, and the abnormality index AI =2.6255. Figure 6c–e show the realizations under mild wind, with $p_{f}=0.01,0.03$ and 0.1, respectively. In Figure 6c, the highest point appears at $\left(x_{c},y_{c},\zeta_{c}\right)=\left(0,0,1.41\right)$, the wave height is $H_{\mathrm{cr}}=2.29$ m, and the abnormality index is AI =2.2967. In Figure 6d, the highest point rises to $\left(x_{c},y_{c},\zeta_{c}\right)=\left(0,0,2.75\right)$ and the wave height and the abnormality index increase to $H_{\mathrm{cr}}=3.89$ m and AI =3.9490, respectively. In Figure 6e, the highest point rises farther to $\left(x_{c},y_{c},\zeta_{c}\right)=\left(0,0,5.05\right)$ and the wave height and the abnormality index further increase to $H_{\mathrm{cr}}=6.27$ m and AI =6.6075, respectively.

### 4.1. Radar Parameters for Rogue Wave Reconstruction and Performance Indices

Table 2 lists the parameters adopted to reconstruct the XTI-SAR image of the sea-surface profile shown in Figure 6. Three sets of parameters are fine-tuned on separate the cases listed in Table 1. To handle cases 1 and 2 of Table 1, the parameters in case a of Table 2 are adopted, which are comparable to those of a Ka-band InSAR ocean topography mission of Surface Water and Ocean Topography (SWOT) [51]. The ground range resolution and azimuth resolution are adjusted to (Δx,Δy)=(2,2) m to acquire a rogue wave at a spatial scale of 200 m. Two look angles, $\theta_{l}=4^{\circ }$ and $\theta_{l}=45^{\circ }$, are selected to discuss the aftermath of the layover effect on rogue wave reconstruction, which will be elaborated in Section 4.3. An appropriate baseline length is selected to ensure sufficient vertical accuracy without exceedingly sophisticated phase images that may cripple the phase unwrapping process. The effect of baseline length will be elaborated in Section 4.4.

The height variation of rogue waves in cases 3 and 4 of Table 1 is milder and requires higher height accuracy. The parameters in case b of Table 2 are adjusted accordingly, with ground range resolution and azimuth resolution enhanced to (Δx,Δy)=(0.5,1) m. The look angle is determined by processing 80,000 rogue wave realizations, which will be elaborated in Section 4.3. The selection of baseline length will be discussed in Section 4.7. The parameters listed in case c of Table 2 are adjusted to reconstruct rogue waves simulated with the parameters listed in case 5 of Table 1, which are steeper than their counterparts based on cases 3 and 4 of Table 2. A larger look angle of $\theta_{l}=60^{\circ }$ is suggested by reviewing the maximum slope distribution, which will be presented in Section 4.3.

Table 2 lists the parameters adopted to reconstruct the XTI-SAR image of the sea-surface profile shown in Figure 6. Three sets of parameters are fine-tuned on the separate cases listed in Table 1. Adjusted for cases 1 and 2 of Table 1, the parameters in case a of Table 2 are comparable to those of a Ka-band InSAR ocean topography mission of Surface Water and Ocean Topography (SWOT) [51]. The height variation of rogue waves in cases 3 and 4 of Table 1 is milder and requires higher height accuracy. The parameters in case b of Table 2 are adjusted accordingly. The parameters listed in case c of Table 2 are adjusted to reconstruct rogue waves simulated with the parameters listed in case 5 of Table 1, which are steeper than their counterparts based on cases 3 and 4 of Table 1.

Performance metrics are required to evaluate the efficacy of the proposed XTI-SAR imaging method in identifying a rogue wave.

Among the five cases, the rogue-wave peak in Figure 6c is barely discernible, and a secondary peak emerges in the upper-right corner of Figure 6b. A proper threshold will be useful to filter out false peaks and manifest the rogue-wave footprint.

**Figure 7:**
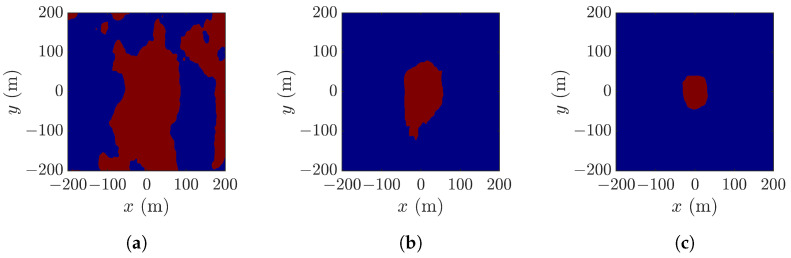
Pixels (red) in Figure 6a with (**a**) ζ>0, (**b**) $\zeta >H_{1/3}/2$, (**c**) $\zeta >H_{1/3}$.

Figure 7 shows the pixels in Figure 6a which meet the thresholds of ζ>0, $\zeta >H_{1/3}/2$ and $\zeta >H_{1/3}$, respectively. Referring to Figure 6a, the threshold $\zeta >H_{1/3}/2$ reveals the rogue-wave peak and footprint, the threshold ζ>0 fails to locate the rogue-wave footprint, and the threshold $\zeta >H_{1/3}$ shrinks the rogue-wave footprint.

**Figure 8:**
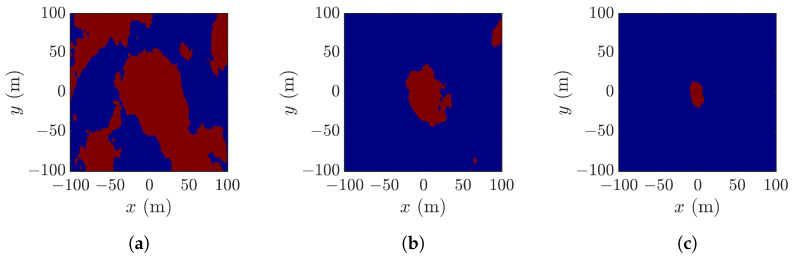
Pixels (red) in Figure 6b with (**a**) ζ>0, (**b**) $\zeta >H_{1/3}/2$, (**c**) $\zeta >H_{1/3}$.

Figure 8 shows pixels in Figure 6b that meet different thresholds. Figure 8b best matches the footprint of a rogue wave, as compared with the other two thresholds.

**Figure 9:**
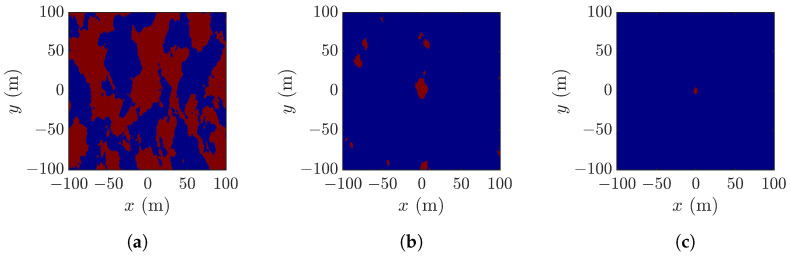
Pixels (red) in Figure 6c with (**a**) ζ>0, (**b**) $\zeta >H_{1/3}/2$, (**c**) $\zeta >H_{1/3}$.

Figure 9 shows pixels in Figure 6b that meet the three thresholds, respectively. No features of a rogue wave can be identified in Figure 9a. Figure 9b manifests the rogue-wave footprint, accompanied by some small speckles. Figure 9c shows small speckle of a possible rogue wave, implying that the threshold $\zeta >H_{1/3}$ is too restrictive to reveal possible rogue waves.

The significant wave height $H_{1/3}$ is the average height of the highest one-third of waves observed in a snapshot. The simulation results indicate that imposing the threshold $\zeta >H_{1/3}/2$ can well reveal an emerging rogue wave and its footprint.

If the true footprint area and the estimated footprint area are *A* and $\tilde{A}$, respectively, a footprint-area error of $\Delta A=\tilde{A}-A$ is used as a performance metric.

Let the true location and peak height of the rogue wave be $\left(x_{c},y_{c}\right)$ and $\zeta_{c}$, respectively, and let their counterparts estimated from the XTI-SAR image be $\left({\tilde{x}}_{c},{\tilde{y}}_{c}\right)$ and ${\tilde{\zeta }}_{c}$, respectively. Then, a peak-location error $\left(\Delta x_{c},\Delta y_{c}\right)=\left({\tilde{x}}_{c}-x_{c},{\tilde{y}}_{c}-y_{c}\right)$ and a peak-height error $\Delta \zeta_{c}={\tilde{\zeta }}_{c}-\zeta_{c}$ are used as performance metrics. The magnitude of peak-location error $\Delta \rho_{c}=\sqrt{\Delta x_{c}^{2}+\Delta y_{c}^{2}}$ is called a peak shift. The difference between the true sea surface ζ and its reconstructed counterpart $\tilde{\zeta }$ over the target area is also quantified by a root-mean-square error (RMSE) as

(27) $$\begin{array}{ccc} & & \mathrm{RMSE}=\sqrt{\frac{1}{N_{x}N_{y}}\sum_{n=1}^{N_{y}}\sum_{m=1}^{N_{x}}{\left(\zeta \left[m,n\right]-\tilde{\zeta }\left[m,n\right]\right)}^{2}} \end{array}$$

### 4.2. Effect of Look Angle

**Figure 10:**
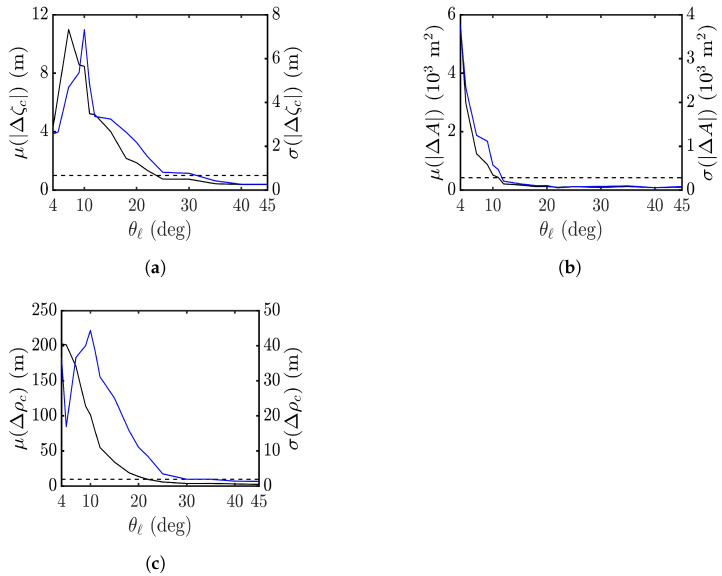
Effect of look angle on (**a**) $|\Delta \zeta_{c}|$, (**b**) |ΔA|, (**c**) $\Delta \rho_{c}$. **———**: average, **———**: standard deviation.

Figure 10 shows the effect of look angle $\theta_{l}$ on the performance metrics in applying the proposed XTI-SAR imaging method to rogue waves, exemplified in Figure 6a. Forty rogue waves are simulated at each look angle of $\theta_{l}$, with $4^{\circ }\leq \theta_{l}\leq 45^{\circ }$.

Figure 10a shows that the average of peak-height error reaches a maximum at $\theta_{l}=7^{\circ }$ and drops below 1 m at $\theta_{l}>25^{\circ }$. The standard deviation follows a similar trend. Figure 10b shows that the average of footprint-area error drops rapidly with the look angle, and ΔA<0.03A if $\theta_{l}>10^{\circ }$. Figure 10c shows that the peak shift $\Delta \rho_{c}$ drops below $0.03\lambda_{p}$ at $\theta_{l}>20^{\circ }$. In short, a more accurate estimation on rogue-wave features is achieved with $\theta_{l}>30^{\circ }$.

### 4.3. Layover Effect and Constraint on Look Angle

**Figure 11:**
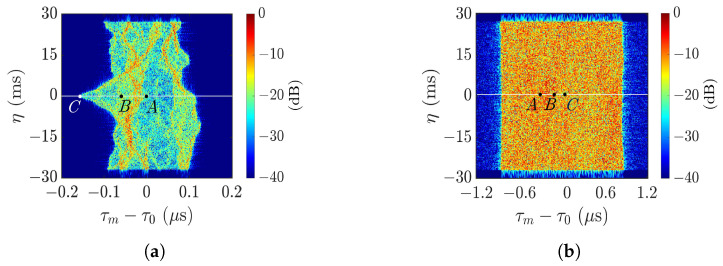
Master SAR images acquired with radar parameters listed in case a of Table 2, (**a**) $\theta_{l}=4^{\circ }$, (**b**) $\theta_{l}=45^{\circ }$.

Figure 11 shows the master SAR images over a rectangular area, acquired by using the radar parameters listed in case a of Table 2, with $\theta_{l}=4^{\circ }$ and $\theta_{l}=45^{\circ }$, respectively. Although a near-nadir look angle renders a higher signal-to-noise ratio (SNR), the surface steepness distorts the SAR image too severely to be used for subsequent XTI-SAR processing.

**Figure 12:**
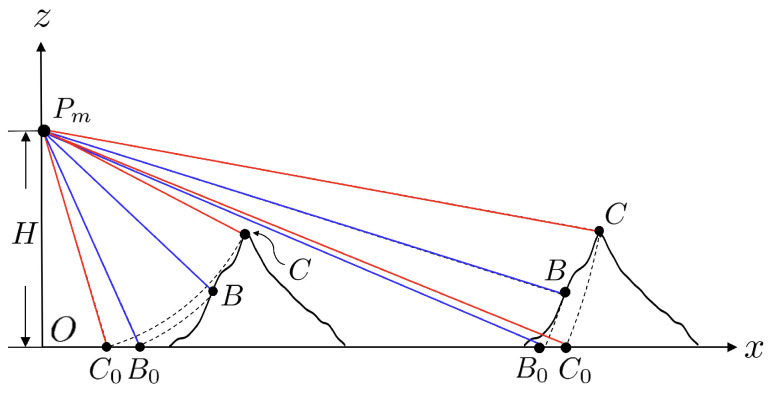
Illustration of layover effect, **———**: *B* is mapped to $B_{0}$, **———**: *C* is mapped to $C_{0}$.

Figure 12 illustrates the layover effect [43] at large look angle (right part) and small look angle (left part), respectively. At large look angle, the backscattered signal from point *C* takes a longer time to reach the receiver than that from point *B*. Thus, point *C* appears at a farther range than point *B* on the reconstructed image. At small look angle, on the other hand, the backscattered signal from point *C* takes a shorter time to reach the receiver than that from point *B*. Thus, point *C* appears at a nearer range than point *B* on the reconstructed image.

**Figure 13:**
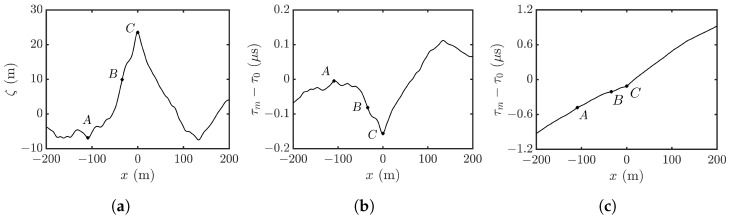
(**a**) Height profile along y=0 in Figure 6a; (**b**,**c**) fast time $\tau_{m}$ of backscattered signal from height profile in (**a**); (**b**) $\theta_{l}=4^{\circ }$, (**c**) $\theta_{l}=45^{\circ }$.

Figure 13a shows the height profile along y=0 in Figure 6c, where points *A*, *B*, and *C* are marked to manifest the layover effect. Figure 13b shows the reconstructed range profile in fast time, with $\theta_{l}=4^{\circ }$. Points *B* and *C* are mapped in reverse order of time, as shown in the left part of Figure 12, leading to the master SAR image shown in Figure 11a. Figure 13c shows the reconstructed profile with $\theta_{l}=45^{\circ }$. The range and fast time are positively correlated, leading to the master SAR image in Figure 11b, which roughly draws a rectangular foundation.

**Figure 14:**
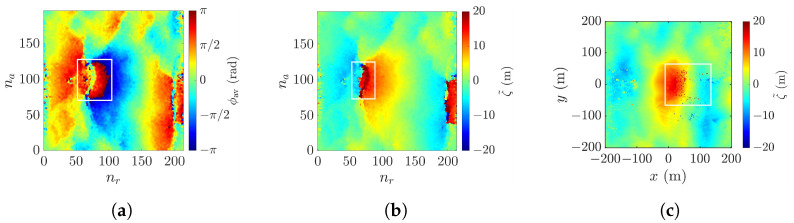
(**a**) $\phi_{\mathrm{av}}\left[n_{r},n_{a}\right]$, (**b**) $\tilde{\zeta }\left[n_{r},n_{a}\right]$, and (**c**) XTI-SAR image of sea-surface profile in Figure 6a, $\theta_{l}=10^{\circ }$, white rectangles mark the regions affected by layover effect.

Figure 14 shows the XTI-SAR image of the sea-surface profile in Figure 6a and two intermediate images, acquired with $\theta_{l}=10^{\circ }$. Figure 14a shows that, within the white rectangle, some pixels around the rogue-wave peak are reversed in range due to the layover effect. Figure 14b shows the estimated sea surface height $\tilde{\zeta }\left[n_{r},n_{a}\right]$ before geometric correction. A bunch of high-elevation pixels marking the rogue-wave peak, as enclosed by the white rectangle, are laterally misplaced. After implementing geometric correction, Figure 14c shows that some pixels with overestimated height are still misplaced, manifesting the artifact speckles enclosed by the white rectangle. This implies that the layover effect is not completely overcome with $\theta_{l}=10^{\circ }$.

**Figure 15:**
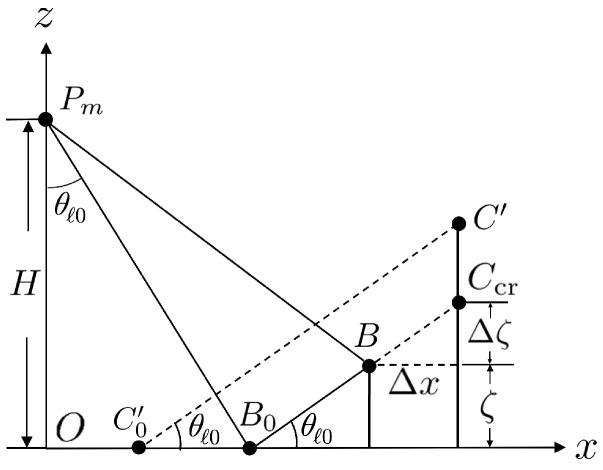
Schematic of maximum steepness allowed at given look angle.

Figure 15 shows the schematic of maximal steepness of rogue waves that can be reconstructed under specific look angle $\theta_{l0}$, without incurring a layover effect [52]. Consider points *B* and *C* that are separated by Δx in the *x* direction and Δζ in the *z* direction. If the slope of $\bar{BC}$ satisfies

$$\begin{array}{ccc} & & \;\frac{\Delta \zeta }{\Delta x}=\tan\theta_{l0} \end{array}$$

then points *B* and *C* have the same range to the radar and will be mapped to the same point in the acquired SAR image. If $\Delta \zeta /\Delta x>\tan\theta_{l0}$, point *C* will be mapped closer to the radar than point *B*, causing a layover effect. Hence, to avoid layover effect under the maximum sea-surface slope of $\xi_{\max}$, the look angle $\theta_{l}$ should satisfy

$$\begin{array}{ccc} & & \;\theta_{l}>\tan^{-1}\xi_{\max} \end{array}$$

**Figure 16:**
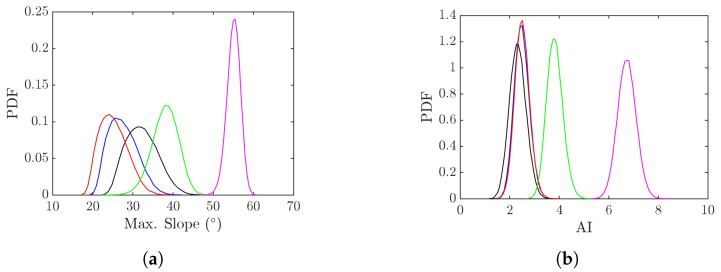
PDF of (**a**) maximum slope (**b**) abnormality index (AI) derived from 80,000 rogue-wave realizations with the parameters listed in Table 1, **———**: case 1, **———**: case 2, **———**: case 3, **———**: case 4, **———**: case 5.

Figure 16a shows the probability density function (PDF) of maximum slope, $\tan^{-1}\xi_{max}$, derived from 80,000 realizations of rogue waves simulated with the parameters listed in Table 1. The maximum surface slope decreases from cases 1, 2, to 3, correlating with the decrease of wind speed, as milder wind tends to drive smoother sea surfaces. The maximum surface slopes of realizations in cases 1, 2, and 3 are below 45°, hence the look angle of $\theta_{l}=$ 45° seems proper.

The maximum surface slope rises significantly from cases 3, 4, to 5, as $p_{f}$ is increased while wind speed $U_{10}$ is fixed. In case 4, the maximum surface slope of most realizations lies below 45°, so adopting $\theta_{l}=$ 45° seems reasonable. In case 5, however, the maximum surface slope of most realizations falls within 50° and 60°; hence, the look angle is adjusted to $\theta_{l}=$ 60°.

Figure 16b shows the abnormality index (AI) derived from 80,000 realizations of rogue waves simulated with the parameters listed in Table 1. The AI is defined as the normalized rogue-wave height with respect to $H_{1/3}$. It is observed that the distributions of cases 1, 2, and 3 are clustered together, and those of cases 4 and 5 shift rightwards. This is consistent with the choice of $p_{f}$, which is the same in cases 1, 2, and 3, and increases in cases 4 and 5. Note that the distributions in all five cases appear symmetric about the peak value.

### 4.4. Effect of Cross-Track Baseline

**Figure 17:**
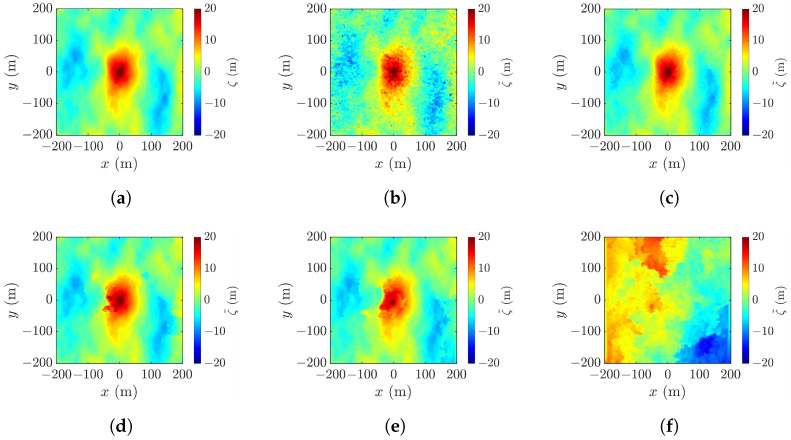
Sea-surface profiles, (**a**) simulated with parameters listed in case 1 of Table 1, reconstructed with (**b**) $b_{⊥}=10$ m, RMSE = 1.77 m, (**c**) $b_{⊥}=200$ m, RMSE = 0.405 m, (**d**) $b_{⊥}=800$ m, RMSE = 0.695 m, (**e**) $b_{⊥}=1200$ m, RMSE = 1.38 m, (**f**) $b_{⊥}=2000$ m, RMSE = 7.17 m.

Figure 17a shows a realization of the sea surface simulated with the parameters listed in case 1 of Table 1, and Figure 17b–f show the reconstructed images using the radar parameters listed in case a of Table 2, with baseline varied from 10 m to 2000 m. Figure 17b shows the XTI-SAR image acquired with short baseline. The features of rogue wave are captured, sputtered with irregular speckles. The peak-height error is $\Delta \zeta_{c}=4.01$ m, the peak-location error is $\left(\Delta x_{c},\Delta y_{c}\right)=\left(5,8\right)$ m, the footprint area error is ΔA=358 m^2^, and RMSE =1.77 m.

Figure 17c shows that, by increasing $b_{⊥}$ to 200 m, the image becomes smoother, the peak-height error is reduced to $\Delta \zeta_{c}=0.27$ m, the peak-location error is reduced to $\left(\Delta x_{c},\Delta y_{c}\right)$ = (3, 3) m, the footprint area error is reduced to ΔA=−199 m^2^, and RMSE is reduced to 0.405 m.

The image acquired by increasing $b_{⊥}$ to 800 m is shown in Figure 17d. The shape of the rogue wave begins to distort, accompanied by the increase of RMSE to 0.695 m. Figure 17e shows that, with $b_{⊥}=1200$ m, the footprint of the rogue wave begins to cave in, the peak-height error is increased to $\Delta \zeta_{c}=-3.34$ m, and RMSE is increased to 1.38 m. The distortion gets worse if $b_{⊥}$ keeps increasing. As shown in Figure 17f, with $b_{⊥}=2000$ m, rogue wave features are not discernible.

**Figure 18:**
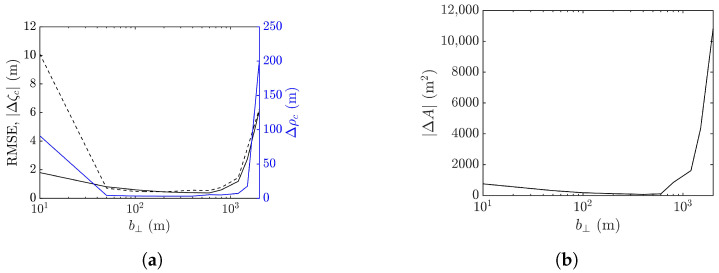
Effects of baseline on performance metrics averaged over 10 realizations of rogue waves based on parameters listed in case 1 of Table 1, (**a**) **———**: RMSE, **———**: $\Delta \rho_{c}$, **- - - - -**: $|\Delta \zeta_{c}|$ (**b**) |ΔA|.

Next, the workable range of the baseline is explored in terms of the performance metrics averaged over 10 realizations of sea-surface profile, as shown in Figure 18. It is observed that the baseline dependence of RMSE, peak shift $\Delta \rho_{c}$, peak-height error $|\Delta \zeta_{c}|,$ and footprint area error |ΔA| are similar. The errors drop as $b_{⊥}$ is increased from a small value, followed by a valley spanning in $b_{⊥}=50$–600 m, over which speckles are suppressed, as in Figure 17b,c. The errors begin to rise with the further increase of $b_{⊥}$, accompanied by the erosion of the rogue-wave peak.

The minimum values of averaged $|\Delta \zeta_{c}|$ and |ΔA| occur at $b_{⊥}=$ 200 m, while those of averaged $\Delta \rho_{c}$ and RMSE occur at $b_{⊥}=$ 400 m. Since the accurate estimation of peak height is more important than the other features of rogue waves, $b_{⊥}=200$ m is more favored than $b_{⊥}=400$ m when the proposed XTI-SAR imaging method is applied to detect rogue waves.

**Figure 19:**
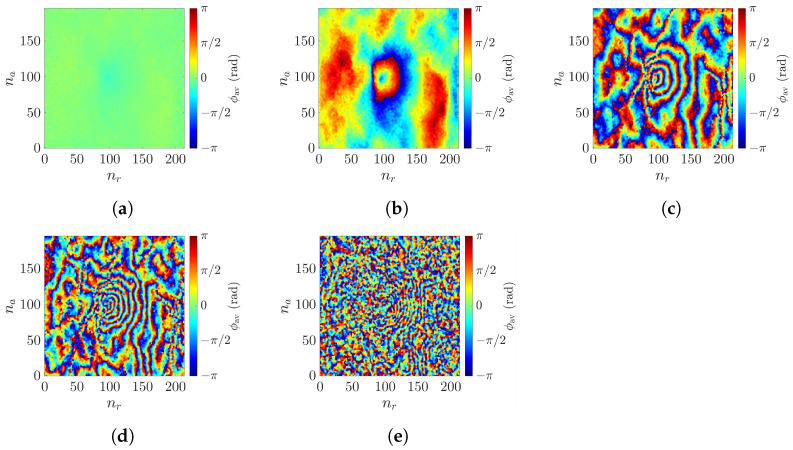
Wrapped phase $\phi_{\mathrm{av}}\left[n_{r},n_{a}\right]$ of the sea-surface profile in Figure 6a, acquired with radar parameters listed in case a of Table 2, (**a**) $b_{⊥}=10$ m, (**b**) $b_{⊥}=200$ m, (**c**) $b_{⊥}=400$ m, (**d**) $b_{⊥}=1200$ m, (**e**) $b_{⊥}=2000$ m.

It was mentioned in [53] that adopting an extremely long baseline would lead to overly dense fringes, frustrating conventional phase unwrapping algorithms. To check if this phenomenon accounts for the increase of RMSE at the long baseline in Figure 18, the phase images $\phi_{\mathrm{av}}$ associated with Figure 17, before phase unwrapping, are shown in Figure 19.

With a short baseline of $b_{⊥}=10$ m, Figure 19a indicates no phase ambiguity, but the resulting height accuracy is low. Discernible features of rogue-wave peak emerge in Figure 19b, accompanied by mild fringes. Figure 19c manifests denser fringes as the baseline is increased to 400 m.

Figure 19d shows that the fringes around the spot with maximum slope turn to squash together, which frustrates the phase unwrapping process and accounts for the distortion observed in Figure 17e. The phase image gets more intricate by adopting an even longer baseline, as shown in Figure 19e; phase unwrapping fails and results in the erratic surface profile in Figure 17f.

### 4.5. Geometric Correction

**Figure 20:**
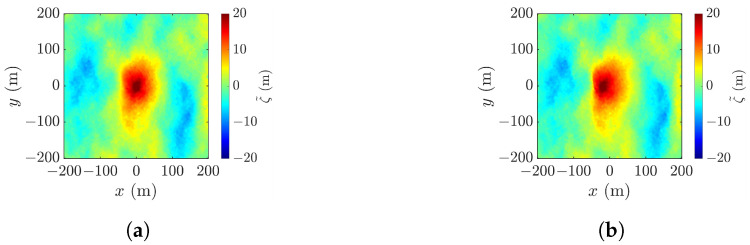
XTI-SAR image of Figure 6a acquired with radar parameters listed in case a of Table 2, (**a**) with geometric correction, ${\tilde{x}}_{c}=-20$ m, (**b**) without geometric correction, ${\tilde{x}}_{c}=$ 3 m.

Geometric correction is not critical in conventional XTI-SAR imaging on sea-surface profiles, because the height-induced geometric distortion is not obvious under most circumstances. However, this is not the case in rogue-wave detection. Figure 20 shows the acquired sea-surface profiles with and without geometric correction, respectively. The rogue-wave peak appears at $\left({\tilde{x}}_{c},{\tilde{y}}_{c}\right)=\left(-20,-3\right)$ without geometric correction and at $\left({\tilde{x}}_{c},{\tilde{y}}_{c}\right)=\left(3,2\right)$ with geometric correction. The corresponding peak-height errors are 0.211 m and 0.271 m, respectively, and the footprint area errors are −198.0 m^2^ and −200.0 m^2^, respectively. This implies that the peak shift of an extraordinary high wave can be prominent. The RMSE value decreases from 0.981 to 0.405 after geometric correction, implying that geometric correction is necessary in rogue wave detection with the XTI-SAR technique.

### 4.6. Effect of Multi-Look Window Size

**Figure 21:**
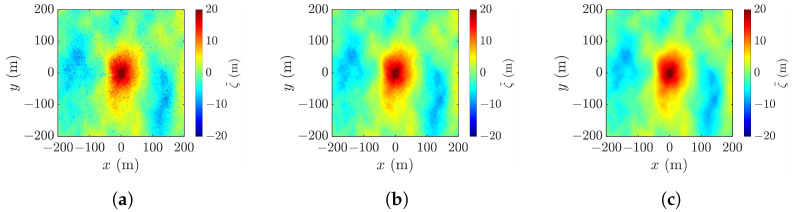
XTI-SAR image of Figure 6a acquired with radar parameters listed in case a of Table 2, (**a**) $N_{wr}=N_{wa}=1$, $\Delta \zeta_{c}=$ 1.88 m, (**b**) $N_{wr}=N_{wa}=3$, $\Delta \zeta_{c}=$ 0.27 m, (**c**) $N_{wr}=N_{wa}=5$, $\Delta \zeta_{c}=-$0.66 m.

After removing the flat-Earth phase, a mean filter of window size $N_{wr}\times N_{wa}$ is imposed on the resulting image to mitigate the phase noise. Figure 21 shows the reconstructed sea-surface profiles of Figure 6a after imposing mean filters of different window sizes. The speckles manifested in Figure 21a are suppressed in Figure 21b, filtered with $N_{wr}=3$ from $\Delta \zeta_{c}=1.88$ m to $\Delta \zeta_{c}=0.27$ m, but is increased to $\Delta \zeta_{c}=-0.66$ m with $N_{wr}=N_{wa}=5$ in this realization.

**Figure 22:**
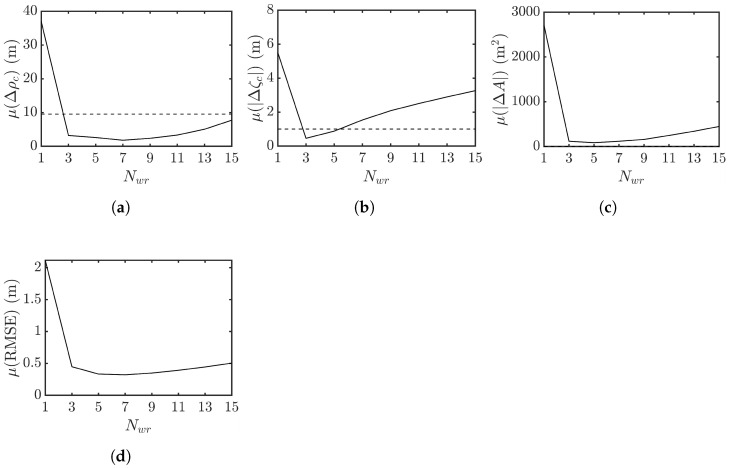
Effect of mean filter size $N_{wr}$ on performance metrics averaged over 10 realizations of rogue wave, $N_{wa}=N_{wr}$, (**a**) $\Delta \rho_{c}$ (**b**) $|\Delta \zeta_{c}|$ (**c**) |ΔA|, (**d**) RMSE.

Figure 22 shows the effect of window size $N_{wr}$ on the mean values of $\Delta \rho_{c}$, $|\Delta \zeta_{c}|$, |ΔA|, and RMSE, respectively, averaged over 10 realizations of rogue waves. Figure 22a,c,d show that the mean values of $\Delta \rho_{c}$, |ΔA| and RMSE drop significantly as $N_{wr}$ is incremented from 1 to 3, vary slightly as $N_{wr}$ is incremented to 7, then rise more obviously as $N_{wr}$ is further increased. Figure 22b shows that the mean value of $|\Delta \zeta_{c}|$ drops significantly as $N_{wr}$ is incremented from 1 to 3, then rises linearly with $N_{wr}$. Since peak height is the key feature of rogue waves, the window size of $N_{wr}=N_{wa}=3$ is suggested for XTI-SAR imaging for rogue-wave detection.

### 4.7. Effects of Wind Speed and Focusing Wave Ratio

**Figure 23:**
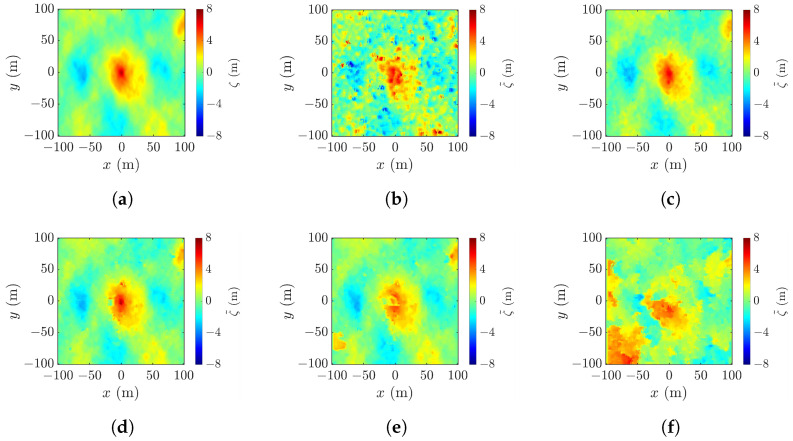
Sea-surface profiles, (**a**) simulated with parameters listed in case 2 of Table 1, reconstructed with radar parameters listed in case a of Table 2, (**b**) $b_{⊥}=10$ m, RMSE = 1.09 m, (**c**) $b_{⊥}=400$ m, RMSE = 0.288 m, (**d**) $b_{⊥}=1000$ m, RMSE = 0.285 m, (**e**) $b_{⊥}=1200$ m, RMSE = 0.486 m, (**f**) $b_{⊥}=2000$ m, RMSE = 1.66 m.

Next, we study the effect of baseline on the reconstruction of various rogue waves characterized with the parameters listed in cases 2 to 5 of Table 1. Figure 23a shows a realization of sea-surface profile with the parameters listed in case 2 of Table 1. The rogue wave has a peak height of $\zeta_{c}=$ 7.08 m and footprint area of A=2794 m^2^. The profile is reconstructed with the radar parameters listed in case a of Table 2, with the baseline varied from $b_{⊥}=10$ m to $b_{⊥}=2000$ m.

Figure 23b shows the reconstructed profile with $b_{⊥}=10$ m. The errors are $\Delta \zeta_{c}=1.60$ m, ΔA=292.1 m^2^, and RMSE =1.03 m. The features of rogue-wave peak are discernible, but sputtered with random speckles, suggesting the baseline is not proper. The peak-location error is $\left(\Delta x_{c},\Delta y_{c}\right)=\left(71,-94\right)$ m, indicating that a spike on the lower right corner is mistaken as the rogue-wave peak. The height accuracy can be improved by adopting a longer baseline, like $b_{⊥}=400$ m. As shown in Figure 23c, the performance metrics are improved to $\Delta \zeta_{c}=-0.72$ m, ΔA=19.8 m^2^, RMSE =0.288 m, and $\left(\Delta x_{c},\Delta y_{c}\right)=\left(5,4\right)$ m.

However, adopting a baseline longer than necessary may have a negative impact. With $b_{⊥}=1000$ m, the footprint of the rogue wave shown in Figure 23d begins to distort, and the error of footprint area is increased to |ΔA|=89.1 m^2^. Figure 23e shows that a further increase of baseline to $b_{⊥}=$ 1200 m renders a blurrier footprint of the rogue wave. Figure 23f shows that rogue waves cannot be recognized with $b_{⊥}=2000$ m.

**Figure 24:**
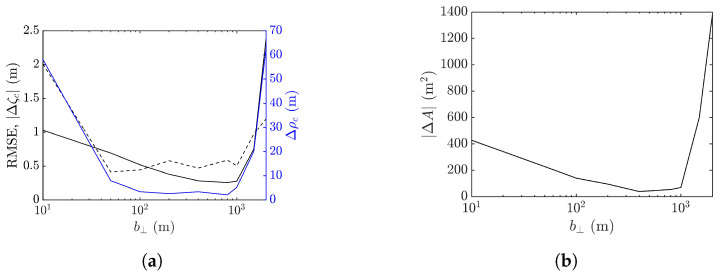
Effects of baseline on performance metrics averaged over 10 realizations of rogue waves simulated with parameters listed in case 2 of Table 1, (**a**) **———**: RMSE, **———**: $\Delta \rho_{c}$, **- - - - -**: $|\Delta \zeta_{c}|$ (**b**) |ΔA|.

Figure 24 shows the performance metrics averaged over 10 realizations of rogue waves simulated by using the parameters listed in case 2 of Table 1. It is observed that RMSE and |ΔA| drop with the increase of baseline to around $b_{⊥}=$ 1000 m, then rise quickly with a further increase of baseline. The peak-height error $|\Delta \zeta_{c}|$ and peak shift $\Delta \rho_{c}$ manifest a valley over $b_{⊥}=50$–600 m, resembling their counterparts in case 1, implying a favorable range of baseline in $b_{⊥}=50$–600 m.

**Figure 25:**
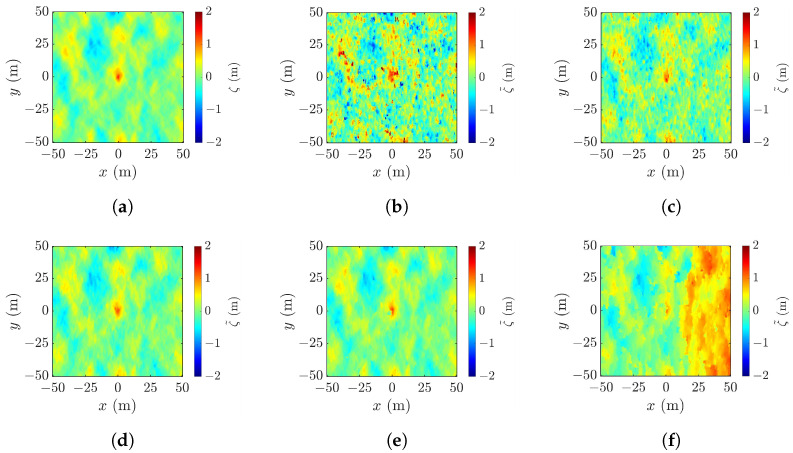
Sea-surface profiles, (**a**) simulated with parameters listed in case 3 of Table 1, reconstructed with radar parameters listed in case b of Table 2, (**b**) $b_{⊥}=10$ m, RMSE = 0.345 m, (**c**) $b_{⊥}=200$ m, RMSE = 0.191 m, (**d**) $b_{⊥}=2000$ m, RMSE = 0.0687 m, (**e**) $b_{⊥}=3000$ m, RMSE = 0.0703 m, (**f**) $b_{⊥}=5500$ m, RMSE = 0.441 m.

Figure 25a shows a realization of sea-surface profiles simulated with the parameters listed in case 3 of Table 1, and Figure 25b–f show the reconstructed images using the radar parameters listed in case b of Table 2, with baseline varied from 10 m to 5500 m. The sea-surface profiles are milder than their counterparts in the previous two cases; thus, the radar parameters are adjusted accordingly.

Figure 25b is overwhelmed by speckles, and the peak-location error of $\left(\Delta x_{c},\Delta y_{c}\right)=\left(5,44\right)$ m indicates failure in capturing the rogue-wave peak. Figure 25c,d show that speckles are suppressed and RMSE values are reduced to 0.191 m and 0.0687 m as the baseline is increased to 200 m and 2000 m, respectively. Figure 25e shows that a further increase of baseline to 3000 m leads to an overly wrapped phase image, similar to that demonstrated in Figure 19e, which fails in the detection of rogue waves, and RMSE is increased to 0.0703 m. Figure 25f shows that, with $b_{⊥}=$ 5500 m, the rogue-wave peak vanishes and the background sea-surface profile is erroneously reconstructed.

**Figure 26:**
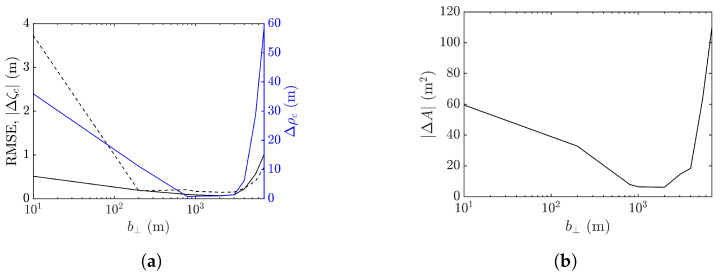
Effects of baseline on performance metrics averaged over 10 realizations of rogue wave simulated with parameters listed in case 3 of Table 1, (**a**) **———**: RMSE, **———**: $\Delta \rho_{c}$, **- - - - -**: $|\Delta \zeta_{c}|$ (**b**) |ΔA|.

Figure 26 shows the performance metrics averaged over 10 realizations of sea-surface profiles simulated with the parameters listed in case 3 of Table 1. The four metrics follow a similar trend: A sharp decrease with baseline, followed by a valley over $b_{⊥}=800$–3000 m, then a sharp rise after that. Judging from the minimum values of these errors, $b_{⊥}=2000$ m is suggested for detecting rogue waves comparable to that exemplified in Figure 6c, accompanied by the radar parameters listed in case b of Table 2.

**Figure 27:**
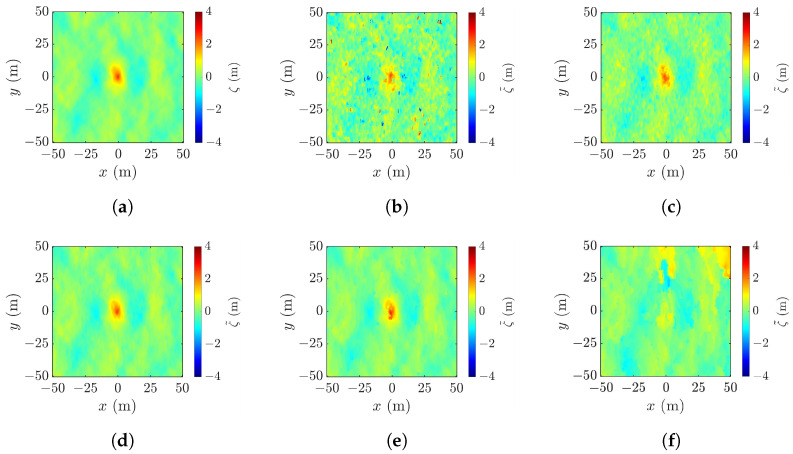
Sea-surface profiles, (**a**) simulated with parameters listed in case 2 of Table 1, reconstructed with radar parameters listed in case b of Table 2, (**b**) $b_{⊥}=10$ m, RMSE = 0.377 m, (**c**) $b_{⊥}=200$ m, RMSE = 0.198 m, (**d**) $b_{⊥}=2000$ m, RMSE = 0.0719 m, (**e**) $b_{⊥}=4000$ m, RMSE = 0.0878 m, (**f**) $b_{⊥}=5500$ m, RMSE = 0.0355 m.

Figure 27a shows a realization of sea-surface profiles simulated with the parameters listed in case 4 of Table 1, in which a larger focusing wave ratio $p_{f}=$ 0.03 is chosen, leading to a sharper rogue-wave peak than its counterpart in case 3. Figure 27b–f show the reconstructed images of Figure 27a using the radar parameters listed in case b of Table 2, with baseline varied from 10 m to 5500 m.

Figure 27b shows, that with $b_{⊥}=10$ m, the image is sputtered with speckles; the errors are $\left(\Delta x_{c},\Delta y_{c}\right)=\left(37.5,43.5\right)$ m, $\Delta \zeta_{c}=3.99$ m, and RMSE =0.377 m. The speckles are suppressed by increasing the baseline to 200 m and 2000 m, as shown in Figure 27c,d, respectively. Figure 27e shows that, by further raising the baseline to 4000 m, the rogue-wave peak begins to vanish. Figure 27f shows that, with $b_{⊥}=5500$ m, the image manifests no trace of rogue waves.

**Figure 28:**
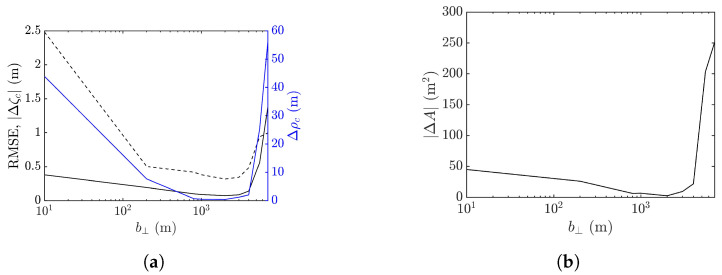
Effects of baseline on performance metrics averaged over 10 realizations of rogue wave simulated with parameters listed in case 4 of Table 1, (**a**) **———**: RMSE, **———**: $\Delta \rho_{c}$, **- - - - -**: $|\Delta \zeta_{c}|$ (**b**) |ΔA|.

Figure 28 shows the performance metrics averaged over 10 realizations of sea-surface profiles comparable to that in Figure 27a. The four metrics appear to display a similar trend: the errors drop with the increase of baseline, then slowly decrease to reach the lowest magnitudes around $b_{⊥}$ = 2000 m, then raise quickly with further increase of baseline.

**Figure 29:**
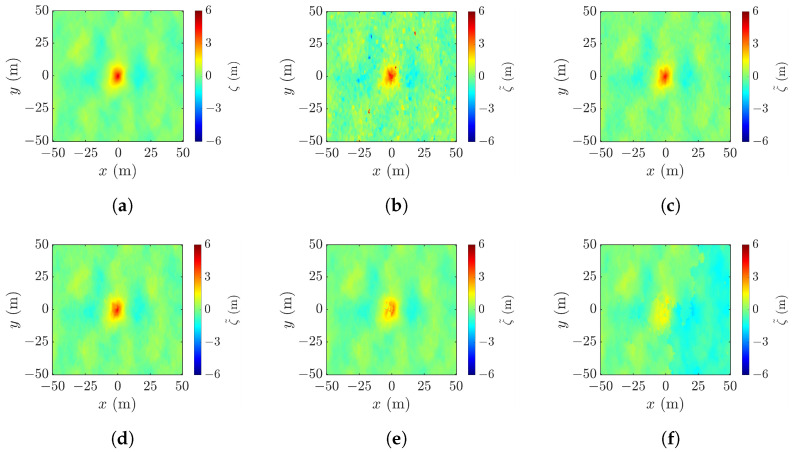
Sea-surface profiles, (**a**) simulated with parameters listed in case 5 of Table 1, reconstructed with radar parameters listed in case c of Table 2, (**b**) $b_{⊥}=10$ m, RMSE = 0.285 m, (**c**) $b_{⊥}=1000$ m, RMSE = 0.113 m, (**d**) $b_{⊥}=2000$ m, RMSE = 0.0903 m, (**e**) $b_{⊥}=4000$ m, RMSE = 0.119 m, (**f**) $b_{⊥}=7000$ m, RMSE = 0.579 m.

Figure 29a shows a realization of sea-surface profiles simulated with the parameters listed in case 5 of Table 1, in which the focusing wave ratio is raised to $p_{f}=0.1$ to generate a distinctively sharp rogue-wave peak. To detect rogue waves with small footprints, a third set of radar parameters are tailored and are listed in case c of Table 2. Figure 29b–f show the reconstructed images with baseline varied from 10 m to 7000 m.

Figure 29b shows the speckled image acquired with $b_{⊥}=10$ m. The errors are $\left(\Delta x_{c},\Delta y_{c}\right)=\left(-24.5,-50\right)$ m, $\Delta \zeta_{c}=-0.11$ m, and RMSE =0.285 m, indicating the rogue-wave peak is not captured. Figure 29c,d show the images with $b_{⊥}=1000$ m and 2000 m, respectively. The speckles are significantly suppressed. However, if the baseline is further increased to $b_{⊥}=4000$ m, the rogue-wave peak begins to vanish. No trace of rogue waves is discernible in Figure 29f, acquired with $b_{⊥}=7000$ m.

**Figure 30:**
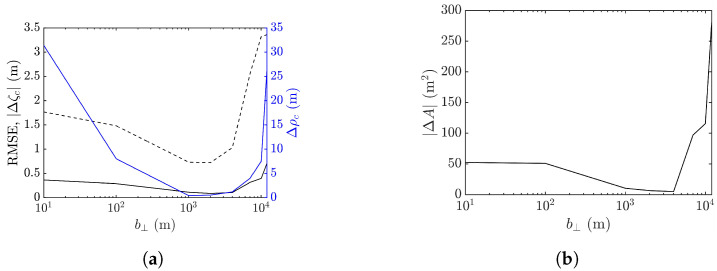
Effects of baseline on performance metrics averaged over 10 realizations of rogue wave simulated with parameters listed in case 5 of Table 1, (**a**) **———**: RMSE, **———**: $\Delta \rho_{c}$, **- - - - -**: $|\Delta \zeta_{c}|$ (**b**) |ΔA|.

Figure 30 shows the performance metrics averaged over 10 realizations of sea-surface profile using the parameters listed in case 5 of Table 1. Judging from the minimum values of these errors, $b_{⊥}=2000$ m is suggested for detecting sharp and narrow rogue waves.

Based on these five cases, it is found that selecting a baseline too short leads to sputtered speckles in the acquired image, and selecting a baseline too long leads to an overly wrapped phase image, which cannot be processed correctly. Thus, a proper range of baseline is available for detecting rouge waves of different types, without artifact speckles. In this work, we not only propose an effective XTI-SAR imaging method to reconstruct sea-surface profiles embedding rogue wave but also propose an efficient method to simulate rogue waves, which are elusive and scarce in field measurements, to validate our imaging method and fine-tune the radar parameters.

Basic knowledge on the sea state is required to fine-tune the parameters in real scenarios without a priori information. The simulation results suggest that $H_{1/3}/2$ is an effective threshold to spot a rogue wave. In practice, $H_{1/3}$ could be estimated from significant wave height (SWH) or wind speed [54], where the SWH was estimated by applying an eXtreme Gradient Boosting (XGBoost-SC) model and cumulative distribution function (CDF) matching technique on the Cyclone Global Navigation Satellite System (CYGNSS) data. In [55], a deep-learning-based technique of WaveTransNet was proposed to retrieve SWH from Global Navigation Satellite System Reflectometry (GNSS-R) data. SAR imagery has also been applied to estimate the wind speed by taking the NRCS data acquired at two polarizations [56]. The radar parameters can thus be selected and fine-tuned based on the estimated value of $H_{1/3}$.

As shown in Figure 16, the slope of rogue waves does not exceed $45^{\circ }$ under most circumstances, implying that a look angle of $45^{\circ }$ is proper. As illustrated in Figure 19, the baseline should be properly selected so as not to result in a phase image too entangled to unwrap. The baseline also affects the height of ambiguity (HoA) [57], which should be approximately $H_{1/3}/2$ in order to detect an emerging rogue wave without height ambiguity.

## 5. Conclusions

An XTI-SAR imaging method is proposed for detecting rogue waves. A rogue wave simulation scheme, composed of random wave train and focusing wave train, is implemented to simulate a plethora of rogue waves like the New Year’s wave at high emergence rate. Sea-surface profiles embedding rogue waves are reconstructed with proper phase unwrapping and geometric correction, which is critical to fix the peak location of rogue waves. The radar parameters for rogue wave detection are customized by minimizing the errors of rogue-wave peak location, height, and footprint area over many rogue wave events. Small errors of rogue-wave peak location, height, and footprint area are reduced to the order of meters, decimeters, and 10 m^2^, respectively, at the spatial resolution of 3 m × 3 m. XTI-SAR images of rogue waves in five scenarios that cover different wind speeds and rogue-wave shapes are reconstructed to confirm the efficacy of the proposed method, and a proper range of the baseline is suggested by inspecting these errors.

As for future research directions, wave–wave interactions, wave breaking, and more sophisticated electromagnetic scattering models can be involved to increase the fidelity of rogue wave generation. In this work, a linear superposition model is applied to generate rogue waves with high efficiency. Wave–wave interactions may add stronger nonlinear effects in modulating the steepness of a rogue wave. Wave-breaking mechanisms, which constrain the rogue-wave steepness, may also be included. Rouge waves emerging under very strong winds may be covered with foam, which affects the radar cross-section and deserves further study.

## Figures and Tables

**Table 1:** Parameters of JONSWAP spectrum for simulating rogue waves.

Parameter	Symbol	Case 1	Case 2	Case 3	Case 4	Case 5
wind speed	$U_{10}$ (m/s)	**23**	**13.7**	7	7	7
significant wave height	$H_{1/3}$ (m)	12	4	1	1	1
peak angular frequency	$\omega_{p}$ (rad/s)	0.44	0.70	1.36	1.36	1.36
enhancement factor	γ	2.51	2.51	2.51	2.51	2.51
energy scaling factor	α	0.0129	0.0092	0.0081	0.0081	0.0081
no. of freq. components	$N_{\omega }$	200	200	200	200	200
no. of angular components	$N_{\theta }$	36	36	36	36	36
focusing wave ratio	$p_{f}$	0.01	0.01	0.01	**0.03**	**0.1**

**Table 2:** Radar parameters for XTI-SAR imaging.

Parameter	Symbol	Case a	Case b	Case c
carrier frequency	$f_{c}$ (GHz)	35	35	35
signal bandwidth	$B_{r}$ (MHz)	93.9	375.6	306.7
radar wavelength	$\lambda_{0}$ (cm)	0.86	0.86	0.86
look angle	$\theta_{l}$ (°)	4/45	45	**60**
polarization		vv	vv	vv
squint angle	$\theta_{\mathrm{sq}}$ (°)	0	0	0
platform altitude	*H* (km)	873	873	873
platform velocity	$v_{s}$ (m/s)	7412.4	7412.4	7412.4
range sampling freq.	$F_{r}$ (MHz)	112.7	450.7	368.0
pulse repetition freq.	$F_{a}$ (Hz)	3600	6900	6900
pulse width	$T_{r}$ (μs)	3/1.5	1.5	1.5
range samples	$N_{r}$	4096/512	2048	1024
azimuth samples	$N_{a}$	1024/2048	8192	8192
coherent interval	$T_{a}$ (s)	0.31	0.62	0.88
along-track baseline	$b_{\mathrm{ati}}$ (m)	0	0	0
cross-track baseline	$b_{\mathrm{xti}}$ (m)	200/400	2000	2000
perp. baseline	$b_{⊥}$ (m)	200/400	2000	2000
parallel baseline	$b_{\|}$ (m)	0	0	0
ground range reso.	Δx (m)	2	**0.5**	**0.5**
azimuth reso.	Δy (m)	2	**1**	**1**
oversampling ratio	$N_{s}$	16	16	16
no. of sub-images	$N_{rt}\times N_{at}$	8×8	8×8	8×8
multi-look window	$N_{wr}\times N_{wa}$	3×3	3×3	3×3

## Data Availability

The original contributions presented in this study are included in the article.
